# A comprehensive review on clove (*Caryophyllus aromaticus L*.) essential oil and its significance in the formulation of edible coatings for potential food applications

**DOI:** 10.3389/fnut.2022.987674

**Published:** 2022-09-15

**Authors:** Vinay Kumar Pandey, Rafeeya Shams, Rahul Singh, Aamir Hussain Dar, R. Pandiselvam, Alexandru Vasile Rusu, Monica Trif

**Affiliations:** ^1^Department of Bioengineering, Integral University, Lucknow, India; ^2^Department of Food Technology and Nutrition, Lovely Professional University, Phagwara, India; ^3^Department of Food Technology, Islamic University of Science and Technology, Pulwama, India; ^4^Division of Physiology, Biochemistry and Post-harvest Technology, ICAR–Central Plantation Crops Research Institute, Kasaragod, India; ^5^Life Science Institute, University of Agricultural Sciences and Veterinary Medicine Cluj-Napoca, Cluj-Napoca, Romania; ^6^Animal Science and Biotechnology Faculty, University of Agricultural Sciences and Veterinary Medicine Cluj-Napoca, Cluj-Napoca, Romania; ^7^Department of Food Research, Centre for Innovative Process Engineering (CENTIV) GmbH, Stuhr, Germany

**Keywords:** essential oil, edible coating, antimicrobial activity, food applications, antioxidant

## Abstract

Many studies have demonstrated the use of synthetic preservatives and chemical additives in food is causing poisoning, cancer, and other degenerative disorders. New solutions for food preservation with quality maintenance are currently emerging. As a result, public concern has grown, as they desire to eat healthier products that use natural preservatives and compounds rather than synthetic ones. Clove is a highly prized spice used as a food preservative and for a variety of therapeutic reasons. Clove essential oil and its principal active component, eugenol, indicate antibacterial and antifungal action, aromaticity, and safety as promising and valuable antiseptics in the food sector. Clove essential oil and eugenol are found to have strong inhibition effects on a variety of food-source bacteria, and the mechanisms are linked to lowering migration and adhesion, as well as blocking the creation of biofilm and various virulence factors. This review emphasizes the importance of CEO (clove essential oil) in the food industry and how it can be explored with edible coatings to deliver its functional properties in food preservation.

## Introduction

Clove is the common name for the herb *Eugenia caryophyllata*, belonging to the *Myrtaceae* family. A range of bioactive compounds, including some potent antioxidants and antimicrobials, are present in cloves, which are the dried flower buds of the clove tree (1). Scientists reported that clove essential oil (CEO) is primarily composed of phenylpropanoids namely eugenol and its derivatives, with low amounts of -humulene and -caryophyllene chemical components (2). CEO's biological qualities, which include antioxidant, antibacterial, antiseptic, pesticide, analgesic, and anticarcinogenic activity, make it useful in numerous industries such as food, biomedical, packaging, sanitary, cosmetics, and pharmaceuticals (3). CEO is often used in food as natural preservative, colorant, and a spice (4). Essential oils comprise both labile and volatile substances that dissolve or evaporate easily during processing, usage, and storage, or while added into food or packaging materials, among other conditions, such as low pressures, high temperatures, the presence of light and air, and others (5). Due to its exceedingly volatile and low water-soluble components, such as eugenol, the CEO's antibacterial and antioxidant capabilities are severely limited (6). Encapsulating bioactive substances like essential oils can be an efficient way to protect them from deterioration in harsh environments and be potentially utilized to increase the shelf life of essential oils and provide delivery systems with the controlled release (7).

It has been investigated that many nanoencapsulation strategies for bioactive substances (8). Ionotropic gelation is a very efficient encapsulation process that results in excellent stability, long usable life, higher loading capacity, good water dispersibility, and controlled release of encapsulated bioactive substances (8). The secondary metabolites of aromatic plants that make up essential oils are a complicated mixture. Due to their antioxidant and antibacterial characteristics, spices like clove, mint, oregano, cinnamon, and thyme have been used for millennia as food medicinal plants and preservatives. Numerous researches have now demonstrated the antifungal, antibacterial, antiviral, and anticarcinogenic properties of spice plants. Since it differs from other spices in terms of its high antioxidant and antibacterial capabilities, clove has attracted a lot of interest. A member of the *Mirtaceae* family tree, *Syzygiumaromaticum* (*S. aromaticum*) (alternative name: *Eugenia cariophylata*), sometimes called as clove, is indigenous to the Maluku Islands in eastern Indonesia. The commerce of cloves and the pursuit of this rich spice have increased the economic development of this Asiatic region for decades (9). Clove trees are commonly grown in coastal locations at maximum altitudes of 200 meters above sea level. After 4 years of plantation, the production of flower buds, which is the tree's commercialized section, begins. Before flowering, flower buds are harvested throughout the maturation phase. The collection could be done manually or chemically, with the help of a natural phytohormone that releases ethylene in the vegetal tissue, causing early maturation. Indonesia, India, Malaysia, Sri Lanka, Madagascar, and Tanzania, particularly the island of Zanzibar, are now the major clove producers. Clove is grown in Brazil's northeast region, in the states of Bahia and Valença, Ituberá, Taperoá, Camamu, and Nilo Peçanha, on an estimated 8,000 hectares, generating close to 2,500 tons per year (10).

Clove oil possesses biological qualities like antifungal, antibacterial, antioxidant, and insecticidal capabilities, and has been utilized in cuisine for centuries as a flavoring ingredient and antimicrobial material (11). Clove oil is also used as an antiseptic in oral infection treatment. Mold, yeast, and bacterial growth are inhibited by clove essential oil (12). It was found efficient against *S. enteritidis* and *L. monocytogenes* in cheese and tryptone Soy Broth. Clove essential oil's significant biological and antibacterial properties are due to the high amounts of eugenol it contains. Eugenol and the phenolic components of clove essential oil have both been demonstrated to denature proteins and interact with phospholipids in cell membranes, affecting the growth and permeability of various gram-positive, gram-negative bacteria and numerous yeast strains. In a time-dependent kinetic process called microbial inactivation, the viability of organisms exposed to biocides varies. Inactivation kinetics are influenced by the concentration and type of biocide, the type of microbe, and environmental variables like pH, temperature, and the presence of organic matter. When exposed to organic fluids like serum, blood, proteins, and so forth, certain chemicals that are potent antibacterial agents in the lab often lose their efficacy (11).

Food spoilage caused by microorganisms results in massive food wastage and is a major public health concern (13). A variety of synthetic preservatives are employed to suppress microbe development in foods, resulting in fewer foodborne medical conditions and longer shelf lives for goods (14). However, their usage is being questioned due to health risks and probable rise in the microbial resistance. As a result, it is desirable to develop and use natural, safe, and effective antibacterial compounds (15). The majority of natural antimicrobials (Derived from plants) can effectively reduce foodborne pathogenic bacteria while also prolonging shelf life (16). Furthermore, as is the case in the majority of situations, plants or their extracts are thought to be generally harmless for people. Many essential oils from a variety of plants, such as edible and medicinal herbs, spices, and plants, are safe and have powerful antibacterial activities (17), and they have found use in medicine and food industries (13).

It has been suggested that EOs could be enclosed in micro- and nanoparticles (MPs and NPs), micro- and nano capsules (MCs and NCs), films, or nanocomposite materials. These techniques increase the stability of CEO in aqueous media and, as a result, increase their bioavailability, lessen their harmful effects, give the encapsulated substance a controlled release, shield them from the environment, or cover up their potent odor (18). Due to their larger surface-to-volume ratios and resulting increases in reactivity, micro- and nanocarriers with customized characteristics are of particular interest. Typically, these systems are based on lipids, polymers, or a mix of the two. Additionally, there are some differences between micro- and nanocarriers in terms of how they behave after application, their capacity to penetrate certain biological barriers, their ability to enter cells, and potential tissue reactions, which will influence the choice of one over the other depending on the application. Encapsulation has been utilized to boost the added value of CEO by extending its shelf life, enhancing its physicochemical stability, achieving controlled release, and suggesting new uses. With intriguing results, various encapsulation techniques using a variety of carriers have been created and documented in the literature. The end products may take the form of emulsions, complexes, liposomes, micelles, or particles or capsules. Most studies have concentrated on its stability and bioavailability enhancement and preservation of its valuable biological properties during processing and storage, while others have focused on its action against specific strains of microorganisms (19).

This review article explains the bioactive properties of CEO and along with classification and potential benefits of CEO. Additionally, this article also gives a brief explanation of edible coatings and materials used to prepare edible coatings which carry the properties of essential oils for providing protection to the fruits and vegetables.

## Classification of clove essential oil based on the chemical composition

CEO has over 30 different chemicals, of which eugenol makes up the majority (at least 50%). *Eugenyl acetate*, -*humulene*, and -*caryophyllene* make up the remaining 10–40% (20).

### Eugenol

**Figure 1:**
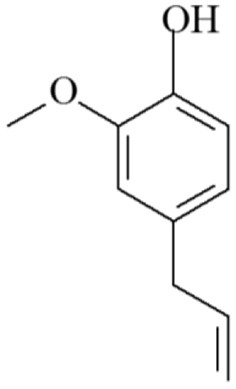
Chemical structure of eugenol.

Clove buds, cinnamon leaves and bark, pepper, turmeric, oregano, thyme and ginger are among the plants that contain eugenol. Several other aromatic herbs, including bay, basil, mace, marjoram, and nutmeg, also contain significant amounts of eugenol. Clove and cinnamon are the most productive plant sources of eugenol, with 45–90% and 20–50% eugenol, respectively. However, the major issues associated with these sources are greater cultivation capital and industrial extraction of eugenol. Tulsi, ginger, bay, and pepper, on the other hand, are inexpensive and plentiful begetters that can be used in place of cloves and cinnamon. Because aerial portions of plants, such as leaves, owers, and bark consist of a significant quantity of essential oils, eugenol is mostly found in these parts. 1, 3 Tulsi leaves also include a high amount of eugenol, usually between 40 and 71%. The eugenol content in various portions of plants, on the other hand, fluctuates with the season. In comparison to summer varieties, studies show that the highest eugenol yield can be produced in the fall season (20). Chemical structure of eugenol is represented in Figure 1.

**Figure 2:**
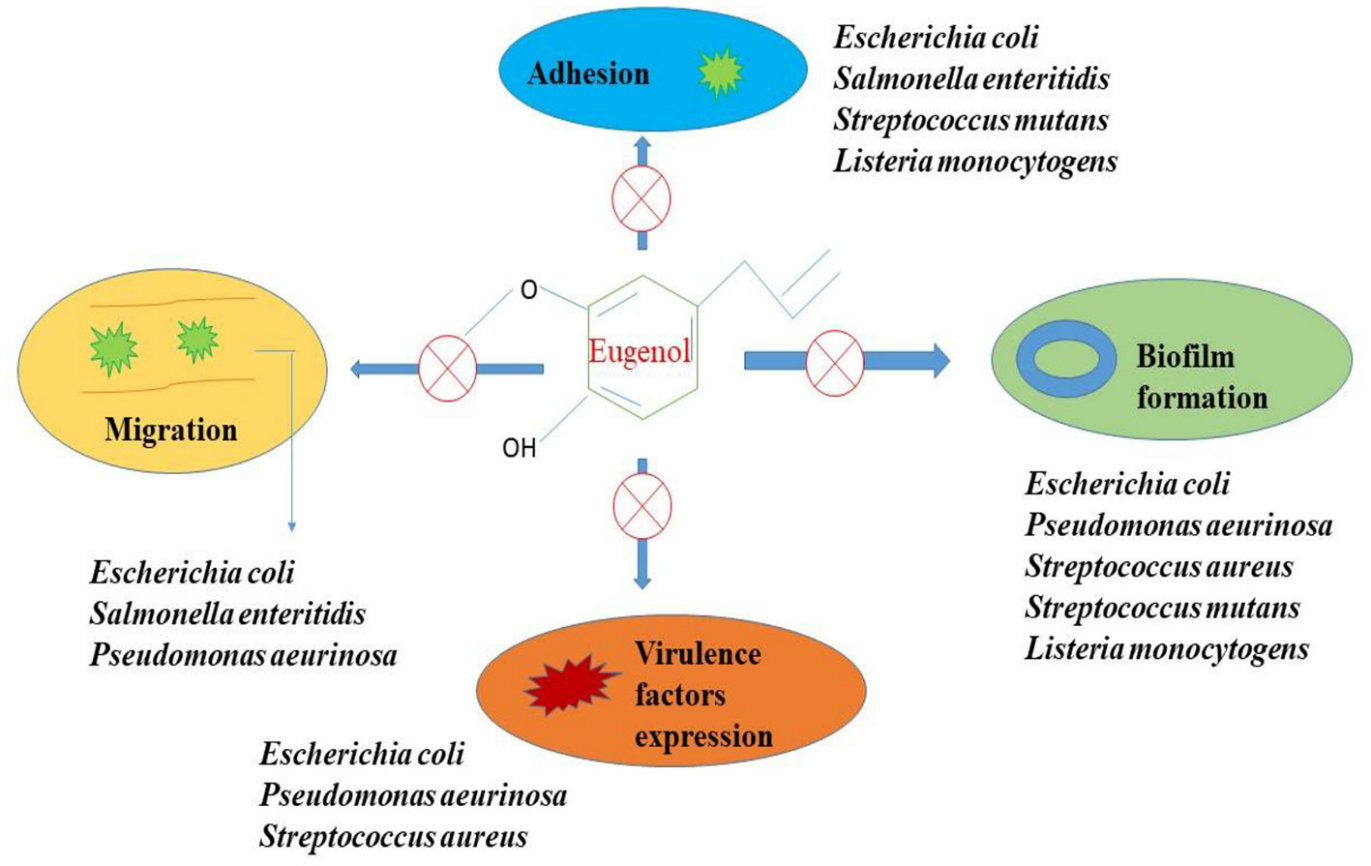
Antibacterial mechanism of eugenol.

*L. Cinnamomum,S. aromaticum, O. vulgare,Z. officinale, P. nigrum, and T. vulgaris* all contain the phenylpropanoid chemical eugenol (21). Approximately 2,460 mg/L of eugenol may be dissolved in water at 25°C, and it has a potent odor and flavor. Eugenol is a volatile molecule that can range in color from colorless to light yellow. Insecticide, antibacterial, anti-inflammatory, antiviral, wound-healing, antioxidant, and anticancer properties of eugenol have all been described (22). In studies using mice, clove oil emulsion was found to possess wound-healing and anti-inflammatory properties (23). Twenty days after the wound, the skins that had been exposed to eugenol displayed reepithelialisation. Eugenol has demonstrated potential anticancer effects against skin, melanoma, leukemia, breast, prostate, stomach, colon, and other cancers (21). Eugenol suppresses the growth and development of tumors, raises the amount of reactive oxygen species (ROS), causes apoptosis, and exhibit genotoxic effects on many cancer cells (24). Antimicrobial mechanism of eugenol is depicted in Figure 2. Eugenol, isoeugenol, and methyl eugenol show insecticidal activity to the storage pathogens, *Sitophilus zeamis* and *Triboliumcostaneum*. The clove leaf and bud oils showed potent insecticidal activity against the human head louse (Pediculus capitis).

### Eugenyl acetate

**Figure 3:**
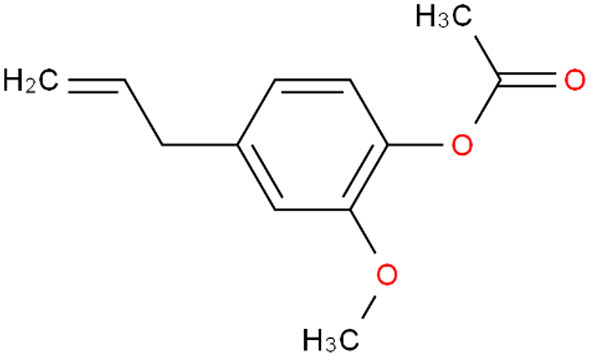
Chemical structure of *Eugenyl acetate*.

*Eugenyl acetate* is a phenylpropanoid derivative of eugenol, possessing antibacterial, antioxidant, antimutagenic, anticancer, and anti-virulence properties (25). It depicted 94.5, 92.1, and 100% inhibition at 200 μg/ml against *H. oryzae, F. moniliforme, and R. solani*, respectively (26). A powerful antioxidant, eugenyl acetate demonstrated 90.30% DPPH free radical scavenging at 35 g/ml and 89.30% NO free radical scavenging at 60 g/ml. However, showed higher antifungal action against Candida spp. and reduced the ability to produce biofilms. At 0.3 g/mL, *eugenyl acetate* likewise demonstrated complete toxicity against *A. salina*. Eugenyl acetate's low fatal doses may also be a sign of toxicity to other microbes, such disease-carrying insect larvae. *Eugenyl acetate* demonstrated potential as a larvicide with an LC50 of 0.1 mg/ml against A. aegypti. The octopaminergic system is primarily interfered with as the mechanism of larvicidal effect. The food and cosmetic sectors have increasing demand because to the advantageous larvicidal, anticancer, antibacterial, and antioxidant qualities (27). Schematic representation of chemical structure is given in Figure 3.

### β-caryophyllene

**Figure 4:**
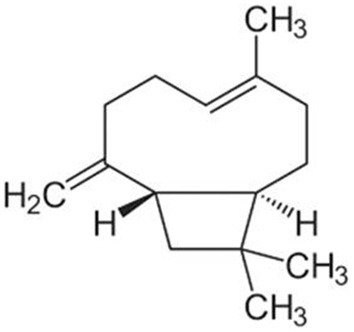
Chemical structure of β*-caryophyllene*.

Clove (*Symphyllum aromaticum L*.), black pepper (*Piper nigrum L*.), hemp (C. sativa L.), *Eugenia cuspidifolia*, guava leaves (*Psidium cattleianum Sabine*), and *Eugenia tapacumensis*, all contain the sesquiterpene -caryophyllene (28). Although the -caryophyllene is soluble in ethanol, it is insoluble in water. Concerning breast, prostate, pancreatic, leukemia, skin, cervical, lymphatic, and cervix cancer, -caryophyllene has shown anti-inflammatory, antibacterial, antioxidant, anticarcinogenic, anxiolytic-like, and local anesthetic actions (29). According to these investigations, -caryophyllene inhibited colon cancer cell growth and proliferation, interfered with tumor formation phases, and lowered the activity of extracellular matrix metalloproteinases. Chemo-sensitizing properties of the -caryophyllene can increase the potency of medications against tumor cells. *An. subpictus* is the most susceptible to -caryophyllene, followed by *Ae. albopictus* and *Cx. Tritaeniorhynchus*. According to the DPPH and FRAP scavenging procedures, the -caryophyllene has a radical scavenging capacity of roughly 1.25 and 3.23 M, respectively. These findings demonstrated the significant antioxidant activity of -caryophyllene. Chemical structure of β*-caryophyllene*is represented in Figure 4.

### α-humulene

**Figure 5:**
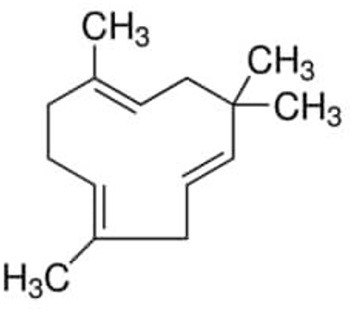
Chemical structure of α*-humulene*.

A sesquiterpene called -humulene can be discovered in *Salvia officinalis L., Senecio brasiliensis,S. aromaticum L*. and *Humulus lupulus*. In lung, prostate, colon, and breast cancer, this substance has demonstrated anti-inflammatory and anticancer properties. According to several findings, the -humulene inhibited colon cancer cell growth and changed the mitochondrial cell membrane (30). Additionally, it can enhance other anticancer bioactivities and the antiproliferative effects of cytostatic medications. According to Fernandes et al. (31), model mice and rats treated orally with -caryophyllene and -humulene (50 mg/kg) had anti-inflammatory effect that were comparable to those of dexamethasone treatment. TNF production is stopped by -humulene, whereas -caryophyllene just slows down its release. Additionally, they lessen the synthesis of prostaglandin E2, nitric oxide synthase, and cyclooxygenase, as well as their inducible expression. Despite having larvicidal action against three vector mosquitoes, *An. Subpictus, Ae. albopictus*, and *Cx. tritaeniorhynchus* (LC50 = 10.26, 11.15, and 12.05 g/ml, respectively), -humulene is safer for *G. affinis* (LC50 = 1024.95 g/ml). Additionally tested against beetle species that prey on stored goods, -humulene demonstrated larvicidal LC50 values of 20.86 g/ml and EC50 values of 77.10 g/ml on *H. armigera* eggs (32). The LC50 value for -humulene's toxicity against *S. granaries* was 4.61 L/mL. At 1 and 3 h after exposure, -humulene decreased *S. granarius'* respiration rate (33). Clove oil can be extracted by different methods depending on the purity concern and need as well (Table 1). Figure 5 represents the chemical structure of α*-humulene*.

**Table 1:** Various extraction methods of clove essential oil.

**Extraction method**	**Principle**	**Advantages**	**Application**	**References**
Solvent extraction	Extraction is carried out using an appropriate organic solvent in a Soxhlet equipment	Yield efficient	Eugenol from tulsi plant leaves using methanol as a solvent and reporting high extraction efficiency	(34, 35)
		Fewer phytochemicals loss		
		Environment friendly		
		Eco-friendly		
		Safety of food		
Microwave-generated hydro distillation (MHD)	The polarity of the microwave influences how much water is soluble in the sample, enhancing the extraction of essential oils	More reliable	Microwave-generated hydro distillation (MGH) in mint leaves has phytochemical stability and yield efficiency	(27, 36)
		Effective Yield		
		Efficient Process		
		Less Volatile loss		
		Less time consumption		
Supercritical fluid extraction (SFE)	Reducing the polarity of complex mixtures into less-polar functions The process permits static and a dynamic model of extraction	Novel process	On-line coupling of SFE to be used as an upright substitute technique for extraction	(34, 37, 38)
		Tuneable solvent power		
		Highly compressible		
		Density can be manipulated with pressure	Determination of various analytes from different samples	
		Eco-friendly		
		Economic		
		High yield		
Ultrasound-assisted extraction (UAE)	The extraction properties with this procedure depend on the matrix of the plant for the quantity and kinetics	Reduce handling time	UAE was implemented for the Essential Oils of rice bran, apricot and almond	(37, 39)
		Less cost of processing		
		Guarantee for the safety of food		
		Efficient yield		
		Proficient		
		Artless		
		Economic substitute		
Microwave-assisted extraction (MAE)	Microwave energy consists of nonionizing radiation with frequencies ranging from 300 to 300,000 MHz that aims at molecular movement by dipolar rotation and ionic polarization	Less degradation of biologically active compounds	Isolation of secondary metabolites of various plant species	(34, 40)
		Reduced extraction time		
		Less solvent consumption		
		Isolation of upgrading yield.		
Hydro distillation	Under the ideal conditions for extracting time, microwave power, and water/plant material ratio, microwave aided hydrodistillation was performed	The hydro-distillation extraction technique has the benefit of not using pricey organic solvent.	With minor modifications in the type of solvent employed and the equipment configuration, this technique is frequently used to isolate volatile and non-volatile polar components from aromatic plants.	(40, 41)
		However, the procedure includes a stage of separation to separate the liquid extract from the water.		
		To promote separation, this processing phase could need to go on for a lengthy time or include using an additional solvent.		

### Iso-eugenol

Eugenol extracted from clove leaf oil can be isomerized to create iso-eugenol. Iso-eugenol (C_1_0H_12_O_2_) has the chemical formula 1-hydroxy-2-methoxy-4-propenylbenzene, 4-hydroxy-3-methoxy-1-propenylbenzene, and 2-methoxy. 4-propenylguaiacol and 4-propenylphenol. It was previously recognized that the commercial precursor for synthetic vanillin (C_8_H_8_O_3_), 3-methoxy-4-hydroxybenzaldehyde, was iso-eugenol. Since isoeugenol contains a phenol moiety, it may undergo conjugation to produce a hydrophilic metabolite. In fact, after oral administration to rats, the phenolic sulfate and glucuronide of isoeugenol are found in the urine (42). As the sulfate, significant levels of side chain oxidized metabolites, including 3′-hydroxylated isoeugenol, are also found. After side chain oxidation, isoeugenol's 3′-position is not susceptible to forming the quinone methide. Therefore, it is not anticipated that isoeugenol will be inactivated by the synthesis of quinone methide to the same degree as eugenol. Cigarettes, non-alcoholic beverages, ice cream, and chewing gum are the main uses for iso-eugenol. Iso-eugenol is mostly produced in Indonesia by tiny distilleries in the Java, Sumatera, Sulawesi, and Bali regions (43).

### Methyleugenol

A number of herbs, including basil, clove, and nutmeg, contain *methylleugenol* (3,4-dimethoxyphenyl-3′-propene), an O-methylated derivative of eugenol, as a bitter taste component. In rodents, *methyleugenol* exerts an anesthetic effect (44). Methyl eugenol occurs naturally in a variety of spices, including nutmeg, clove oil, and others. Numerous studies have demonstrated that methyl eugenol is not one of the main components of clove oil and only appears in extremely small concentrations (less than 1%) (45). Only extremely small amounts, or even a few drops, of clove oil are used in food, ensuring that the amount of methyl eugenol present is negligibly low.

## Extraction methods of clove essential oil

CEO can be extracted through various methods that are explained below.

### Hydro distillation

**Figure 6:**
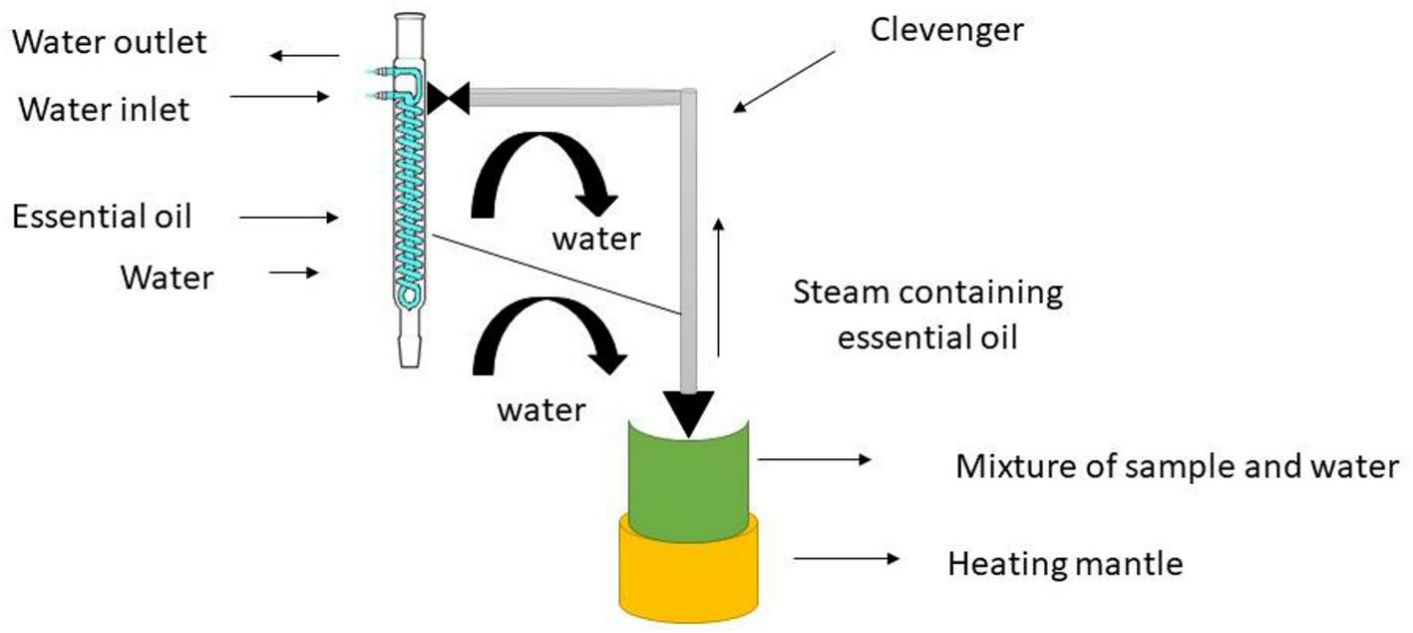
Process of hydro distillation.

Hydro distillation (HD) and organic solvent extraction are two common traditional CEO extraction techniques that typically have the disadvantage of poor work efficiency, high cost, and significant pollution. Nowadays, with the advancement of technology and the green concept, some novel methods have been developed to address the drawbacks of traditional extraction strategy. Examples include microwave-assisted hydro distillation (MAHD) ultrasound-assisted hydro distillation (UAHD), and enzyme-assisted hydro distillation (EAHD). The essential oil from *Aquilaria malaccensis* wood was extracted using a Clevenger-style device, as described by European Pharmacopeia. To hasten the release of the essential oil, the wood was submerged in water for seven days before extraction. Then, 100 g of *A. Malaccensis* wood that had been air dried and reduced in size together with the necessary quantity of distilled water were added to a heating mantle that was linked to the Clevenger. To create steam that contained both water and essential oil, the sample mixes were heated to a boil at 100°C and one atm. This substance was concentrated and gathered in a Clevenger jar. During the extraction procedure, the surplus condensed water was recycled into the flask. This is the most straightforward and frequently least expensive method of distillation. Powders and extremely hard materials like roots, wood, or nuts seem to respond best to hydro distillation. The key benefits of this approach include using less steam, processing more quickly, and producing more oil. In distillation, the plant material is heated either by being submerged in boiling water or by being passed through a steam stream. The plant material's cell walls burst and crumble under the heat and steam, releasing the essential oils. The components of the essential oil and the steam are transported *via* a pipe and directed *via* a cooling tank before changing back to liquid form and being deposited in a vat. Since crude oils are not water-soluble, it is simple to separate them from the water and siphon off the resulting liquid, which is a combination of oil and water. The surface of the water will be covered with essential oils that are lighter than it. Schematic representation of the method is given in Figure 6 (21).

### Steam distillation

Distillation is the traditional technique for detachment of volatile substances from plant material to create essential oils. When aromatic plants are subjected to steam or boiling water during distillation, their essential oils are released by evaporation. The restoration of the essential oil is facilitated by the hypothesis that, at boiling temperature, the combined vapor pressures equal ambient pressure when distilling two immiscible liquids, namely water and essential oil. As a result, the constituents of essential oils, which normally boil at temperatures between 200 and 300°C, evaporate at a temperature akin to that of water. The rising steam that contains the essential oils enters the narrow cooling tube. The quantity of essential oil obtained depends on the duration of the distillation process, the operating pressure, the temperature, and most importantly, the quality and type of the plant material. The extraction of essential oils from plants typically ranges from 0.005 to 10%. Three different methods of evaporation have existed historically: water distillation, water-steam distillation, and steam distillation. The process of distilling water is also known as “indirect” steam distillation. This process involves soaking plant material in water and boiling it. Boiling water produces steam, and that steam carries the volatile oils with it. The oil and water are then separated by cooling and humidification (46).

**Figure 7:**
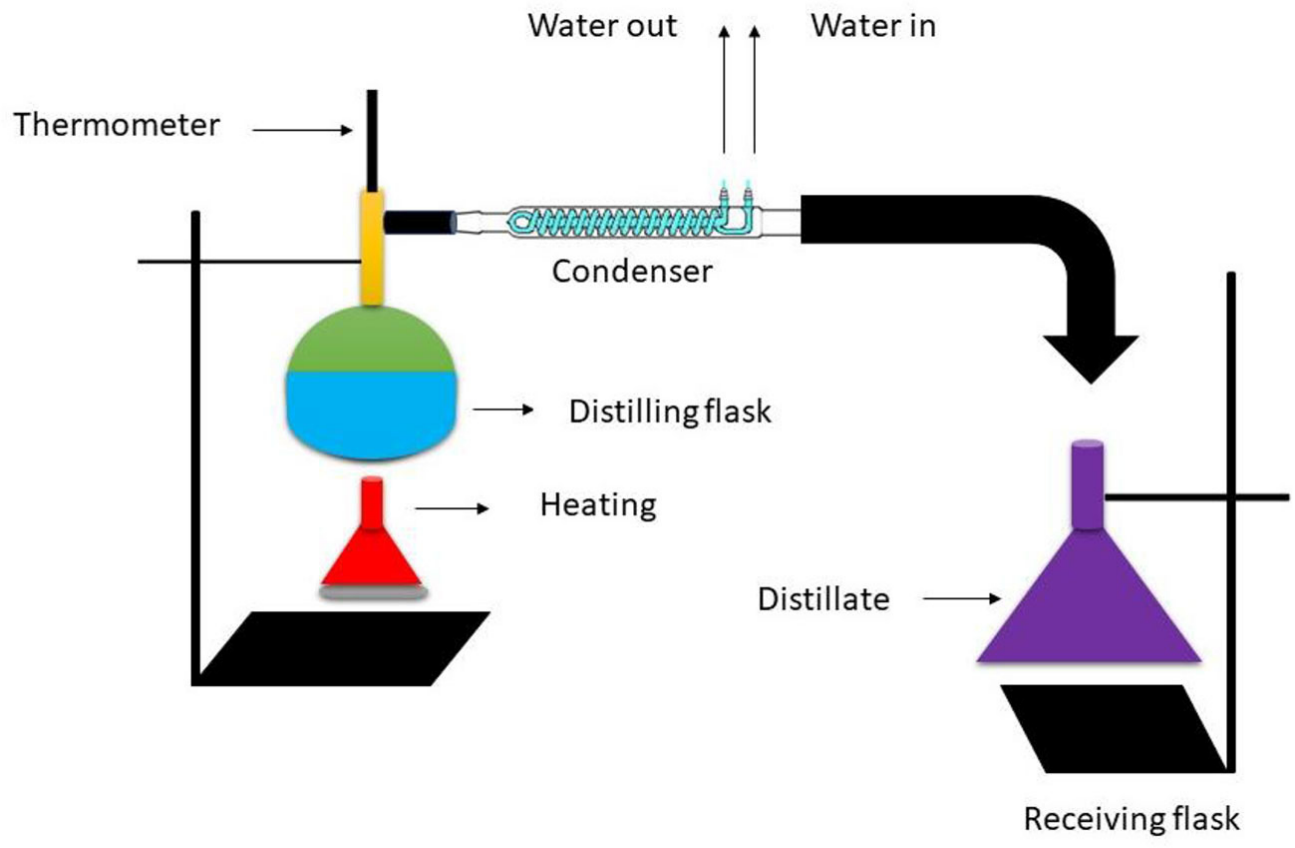
Simple distillation process.

The drawback of this method, aside from its slowness, is that materials and fragrances deteriorate over time when exposed to heat. In the steam method, the leafy plant material is kept on a grill over the hot water, and the steam passes *via* plant material. To ensure even steaming and thorough extraction, the leaves must be placed on the grill with care. The method used to extract essential oils most frequently is “direct” steam distillation. In this procedure, the distillation tank itself does not contain any water. Instead, steam is introduced into the tank from an external source. When the steam ruptures the sacs housing the oil molecules, the essential oils are released from the plant material. At this time, condensation and segregation are standardized processes. In complement to those already mentioned, there are numerous more improved methods for finding organic fragrance components and essential oils, including turbo-distillation, hydro-diffusion, continuous-distillation, vacuum-distillation, cold-expression, molecular-distillation dry-distillation. These conventional extraction techniques all have substantial drawbacks, such as limited yields, contaminant generation, and constrained stability. The steam bubbled through the flask of vegetation in the hydro-distillation and steam distillation processes, evaporating the oil as it goes. As the emerging mixture of vaporized water and oil goes through a coil that is frequently cooled with access to water, the steam condenses there. Increasingly being adopted is used to extract the condensed water and essential oil, or centrifugation in extremely rare cases. Diagrammatic representation is given in Figure 7.

### Super critical fluid extraction

**Figure 8:**
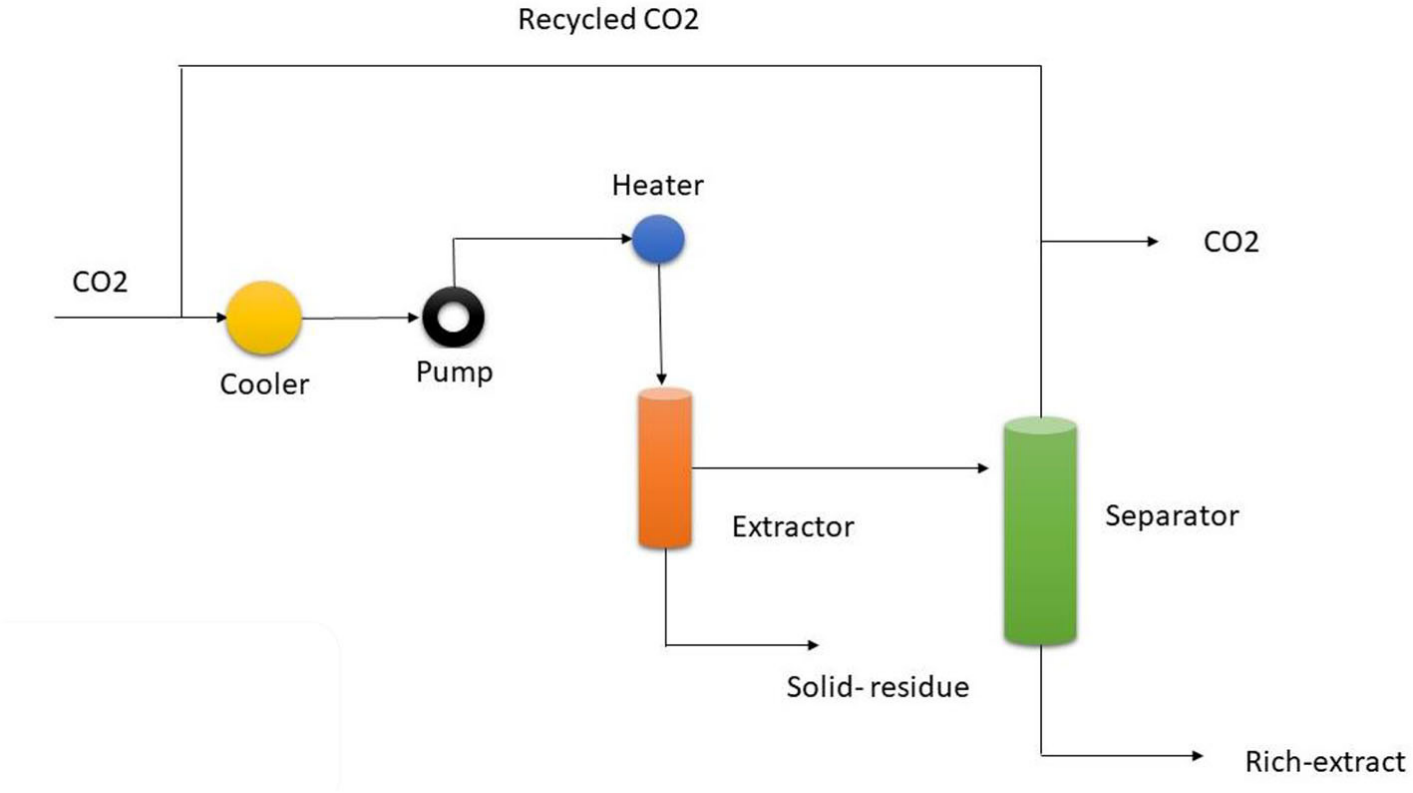
Super critical fluid extraction process.

The use of supercritical CO_2_ (SC-CO_2_), which is non-flammable, nontoxic, noncorrosive, and simple to handle, makes supercritical fluid extraction (SFE) a green technology that can operate at low pressures and close to room temperature. Supercritical CO_2_ (SC-CO_2_) can solubilize lipophilic molecules, and is inexpensive and easily accessible in bulk amounts with a high degree of purity, ensuring minimum change of the bioactive chemicals and keeping their curative or functional characteristics (Figure 8). It is a green and “generally acknowledged as safe” (GRAS) method. Since its inception, the extraction method most commonly referred to as supercritical fluid extraction (SFE) has been praised for its outstanding performance. Currently, SFE is widely used in a variety of fields, including toxicology, chemistry, the environment, textiles, petrochemicals, and polymers, in addition to the food and medicine industries. The extraction of natural plant materials utilizing this method of extraction has been pushed by significant advances in the field of supercritical fluid technology over the past three decades and has been characterized as an environmentally friendly technology. These natural sources could include, among others, plants, algae, and microalgae. Additionally, this method's objectives include using nontoxic organic solvents, low extraction durations, better pollution protection, and good selectivity.SFE is based on certain fluid characteristics including diffusivity, density, dielectric constant, and viscosity, and typically entails changing specific parameters like temperature and pressure to achieve a supercritical fluid. Given these conditions, a fluid exists between both the gas in question and the liquid since the density of the SF is similar to that of the liquid and its viscosity to that of the gas. In other words, the supercritical state of a fluid is the state in which the properties of a liquid and a gas are the same. Additionally, SFs offer greater transportation qualities than liquids because their density, unlike that of liquid solvents, may be changed by adjusting the pressure and temperature. SFE is based on certain fluid characteristics including density, dielectric constant, diffusivity, and viscosity, and typically entails changing specific parameters like pressure and temperature to achieve a supercritical fluid. Given similar conditions, a fluid exists between the gas in question and the liquid since the density of the SF is similar to that of the liquid and its viscosity to that of the gas. In other words, the supercritical state of a fluid is the state in which the properties of a liquid and a gas are the same. Additionally, SFs offer greater transportation qualities than liquids because their density, unlike that of liquid solvents, may be altered by adjusting the pressure and temperature (47).

### Microwave-assisted hydro distillation

**Figure 9:**
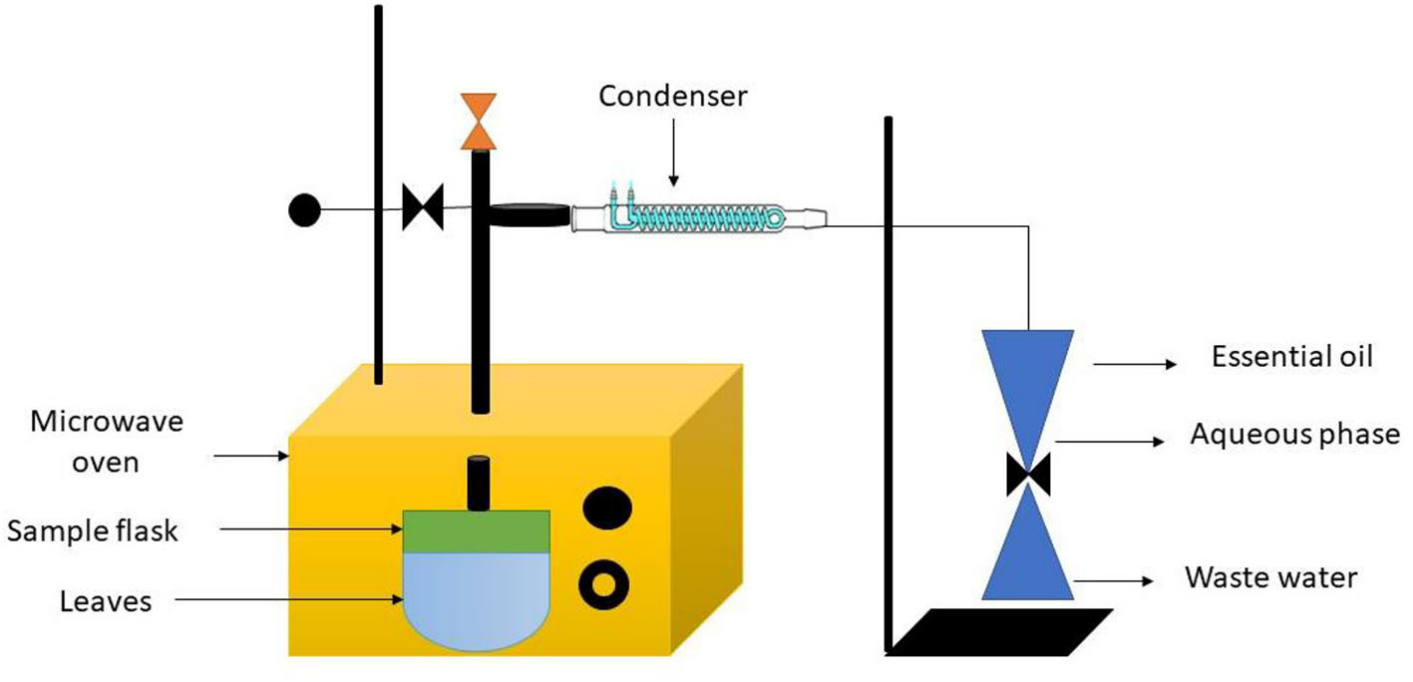
Solvent–free extraction microwave method.

Rapid warming in the microwave field and conventional solvent extraction are combined in the Microwave-assisted Hydro-distillation (MAHD) process. This allows for significant time savings, allowing the recovery to be finished in a matter of minutes. MAHD was created and utilized for the extraction of essential oils from *Xylopia aromatica* (Lamarck) and *Lippia alba* (Mill) in an effort to combine microwave heating with the traditional HD (48). For laboratory scale applications in the extraction of essential oils from various kinds of aromatic plants, Tigrine-Kordjani et al. (49) created a Microwave Assisted Distillation (MAD) using free solvent (Figure 9). Chemat created solvent-free microwave extraction (SFME) in 2015 (50). This procedure, which follows a very straightforward principal, entails the dry distillation of a fresh plant matrix with the use of a microwave without the use of water or any other organic solvent. SFME is not a modified hydro-distillation (HD), which utilizes a lot of water, nor is it a modified microwave-assisted extraction (MAE), which uses organic solvents. The glands and oleiferous receptacles burst as a result of the selective heating of the *in-situ* water content of plant material. Thus, the essential oil is liberated from the plant material and evaporated by azeotropic distillation with the water. To add the original water back to the plant material, the additional water can be refluxed into the extraction vessel. This method has been used on a variety of fresh and dried plants, including citrus fruits, aromatic herbs like basil, mint, and thyme, and spices like ajowan, cumin, and star anise. Sui et al. (51) developed an effective Microwave Pre-treatment (MP) approach to preserve the quality of postharvest rosemary leaves and found that it might be an effective way to preserve quality and extract essential oil from rosemary and other fragrant herbs. At atmospheric pressure, 100 gr of rosemary were heated for 30 min with the addition of 300 ml of water in a conventional MAHD technique. The sample was adequately extracted of all the essential oils during this time. At least three extractions were made for everyone.

### Ohmic heating-assisted hydrodistillation

**Figure 10:**
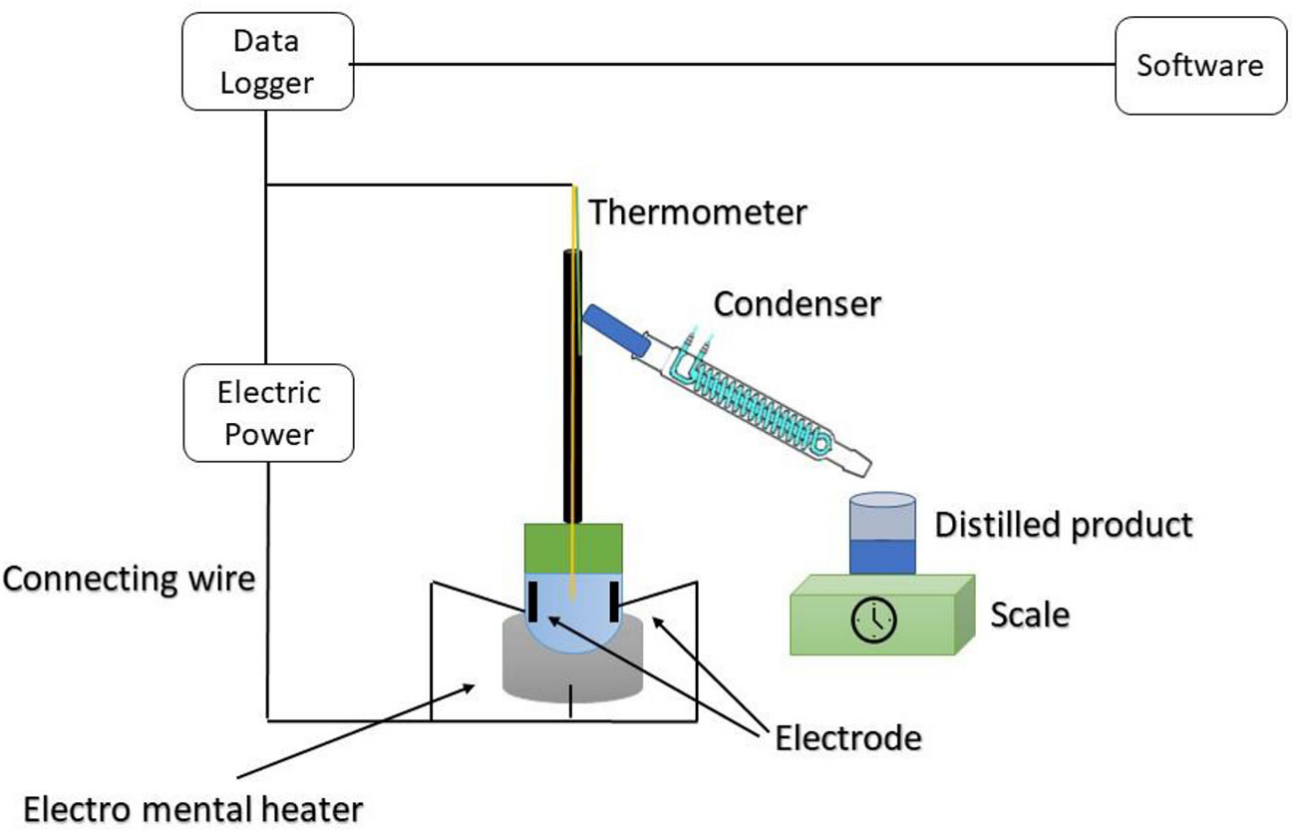
Ohmic heating mechanism.

In comparison to conventional hydrodistillation, ohmic heating assisted hydrodistillation is a revolutionary approach that has promise for the efficient extraction of essential oils from a variety of plant sources (Figure 10). Hydrodistillation with ohmic heating assistance is a green technology that can be scaled up for the manufacture of essential oils. Ohmic heating is the mechanism by which foodstuff produces heat when an electric current is passed through it due to its electrical impedance. Consequently, heat the cell. The two key variables in an ohmic heating process are voltage gradient and electric permeability. Food's electric conductivity is influenced by a number of factors, including the amount of free moisture, ionic strength, and dietary microstructure. The material is subjected to an electric current, which produces heat energy (52). The electricity supply, electrode, and ohmic heating cell are the main components of ohmic heating systems. The two key variables in an ohmic heating process are voltage gradient and electric conductance. Food's solution concentration, free water content, and microstructure are some of the factors that affect a food's ability to conduct electricity. During ohmic heating with a constant voltage gradient, the rate of heat production changes proportionally to electrical conductivity. According to Tunç and Koca (52), ohmic heating technology is more efficient than traditional techniques in terms of uniform heating, working to improve food quality, reducing energy consumption and cost, advancing energy efficiencies, and allowing it to warm food quickly.

### Ultrasound-assisted extraction

UAE involves sending vibrations of ultrasonic energy through with a liquid solution that contains solid particles. A force that is either perpendicular to or comparison to the exterior of the substance is produced as the vibrations impact it. The seismic waves that result from the conversion of sonic energy into mechanical energy have a pressure equivalent to several thousand earth's atmospheres. The rupture of cell membranes, which makes it easier for the fluid to enter the cell and retrieve the marvelous as a result, is thought to be caused by the quick localized rises in pressure and temperature (53). In ultrasound-assisted extraction (UAE), the samples are exposed to mechanical energy produced by ultrasound waves. Small vacuum bubbles or nullifies created by this ultrasonic treatment, known as cavitation, implode at the solid sample, generating localized extreme temps (roughly 4,500°C) and pressures (about 50 MPa). These forces cause sonolysis, the rupturing of cell membranes, and the removal of cellular contents, among other effects. UAE is typically split into direct and indirect UAE. In direct UAE, a sonotrode (an inert acoustical tool) is submerged in the sample-solvent mixture before the ultrasonic irradiation is applied directly. An ultrasonic bath, which can be used for numerous samples at once, is employed to apply indirectly UAE ultrasonic radiation to the sample-solvent mixture. Both strategies need an extra step of clean-up. Temperature, sonication cycle, granulation/grade of homogenization, and extraction time are frequent variables to optimize UAE (54).

## Role of CEO in the food industry

Greater CEO concentrations than those utilized in *in-vitro* experiments are typically needed for complex food matrices. For instance, diets with a high protein content may result in complexes of protein-phenolic EOs that lessen their efficacy (55). Additionally, the antibacterial substance might be absorbed *via* the fat portion of food, which lessens its bactericidal effect. Similarly, lowering the water content in meals may prevent antibiotic drugs from reaching the microbial cell's active site (56). The efficacy of the EO can also be influenced by outside variables including storage temperature, packing, initial concentration, application technique, and the type of microbe (57). Numerous applications of CEO in food industry are mentioned in Table 2.

**Table 2:** Applications and nutraceutical actions of CEO in food industry.

**Category**	**Food**	**CEO Bioactivity**	**Nutraceutical action on foods**	**Dose**	**Application**	**Shelf Life**	**References**
Dairy	Soft Cheese	Antioxidant activity	Inhibit lipid peroxidation	0.01%	Food Fortification	Up to 3 Weeks	(20)
	Cottage cheese		Prevent Protein degradation		Storage		
Baked	Cake	Anti-inflammatory	Prevents inflammatory expressions	1–1.2%	Coating	10–15 days	(58)
	Bread		Inhibit action of prostaglandin synthesis		Storage		
Meat poultry and seafood	Grounded beef	Antimicrobial	Induce cell lysis of gram positive and gram-negative bacteria	0.5–5%	Fortification	7–45 days	(59)
	Shrimp				Coating		
	Chicken patty				Storage		
	Salmon burgers						
	Cod fillets						
	Chicken breast						
Processed food	Ketchup	Neuro-protective	Inhibit lipid peroxidation	500 ppm−2,000 mg/l	Fortification	14 days	(60)
	Sausages	Antistress perspective	Upregulates the concentration of antioxidant enzymes				
			Reduces ulcer stress				
Fruits and vegetables	Mango	Anticancerous activity	Trigger cell apoptosis	0.2–1.56%	Coating	17–21 days	(61)
	Avocado		Reduction of DNA oxidation		Storage		
	Persimmon						
	Pak choi						

### Dairy products

Foodborne disease outbreaks have been linked to the consumption of dairy products like cheese. As an antibacterial agent for cheese manufacturing, Ahmed et al. used roughly 1 kilogram of CEO per 200 litters of raw milk. During a month at 4 degrees Celsius, the CEO showed substantial antibacterial action without compromising organoleptic qualities, indicating a potential cost-effective usage (57). The availability of unique ingredients that can be used to improve the value, quality, natural preservation, shelf life, and novelty of traditional Indian dairy products has expanded as a result of scientific advancements in the fields of food science and technology. Physical-chemical, sensory, anti-oxidant, and microbiological tests were performed on the samples, and the results showed that raising the CEO levels in burfi increased its anti-microbial and anti-oxidative properties. With a rise in CEO addition in burfi, the antioxidant activity rose and the total viable bacterial, yeast, and mold counts significantly decreased. Additionally, the burfi's physicochemical characteristics, including as its chemical composition and instrumental color and texture features, were unaffected by the inclusion of herbal essential oils (62).

### Baked food

The baked goods sector places a strong emphasis on maintaining nutrition and safety standards as well as mold development prevention. Aseptic packaging, irradiation, modified storage environment, and preservative acids are some of the preservation techniques for baked goods. However, due to their detrimental human health effects, organic acids (such as benzoic, propionic, and sorbic acids) are restricted in many nations. Eugenol gives CEO broad-spectrum efficacy against pathogenic bacteria that cause foodborne illness, including *Penicillium species, Aspergillus species, Staphylococcus aureus* and *Escherichia coli*. Baked items can increase their shelf life without changing the original sensory acceptability including taste, appearance, texture, flavor if amalgamated with essential oil (57). When compared to a synthetic antioxidant over the course of 28 days of storage, CEO displayed excellent antioxidant activity and powerful antibacterial qualities. The ratings of the cakes containing CEO were practically on par with the control. The cake sample with 800 ppm of CEO, however, received less favorable reviews than the other samples. Therefore, in addition to having no harmful effects on human health, this essential oil can be used as a natural antioxidant and antibacterial in foods, especially those that include fat, and can extend the shelf life of those items (63).

### Packaging materials

Recently, novel biodegradable packaging materials made of natural polymers have been created (polysaccharides, lipids, proteins). By adding essential oils, their antioxidant and antibacterial capabilities can be improved, expanding shelf life and reducing or inhibiting the growth of foodborne pathogens (64). According to research, the integration of EO into coated films aims to change the functional characteristics, such as permeability of water vapor, antibacterial and antioxidant capabilities (65). Due to CEO compounds' penetration and disintegration of the cell structure, CEO-enriched films demonstrated antibacterial effects and microbial deactivation for up to 21 days (66). By altering the spatial distance inside the film matrix, the incorporation of CEO may change the moisture content of packaging materials, resulting in thicker films. Food look and quality are impacted by the optical characteristics of films. The rate of lipid oxidation is also slowed down by coating application. In this way, the various coloring elements of the CEO can alter the film's hue. Due to a rise in light scattering brought on by oil droplets in the film framework, its integration raises opacity values. These lessen transparency, which is beneficial for foods that are photosensitive. The addition of a CEO to a film network partially substitutes weaker contacts (polymer-oil) for stronger interactions (polymer-polymer). Rearranging the polymers results in a more heterogeneous microstructure and discontinuous network. In addition, the presence of a CEO has a plasticizing impact that lowers the film's elastic modulus the glass transition temperature (67). When CEO was incorporated into films formed from mechanically deboned chicken flesh protein, Saricaoglu and Turhan saw a drop in tensile strength and elastic modulus. As advised for coating film on food by Saricaoglu and Turhan (65), the tensile strength was maintained above 3.5 MPa. Rougher and more porous films were also formed as a result of these structural changes brought about by the addition of the CEO (68). When combined with edible coatings, clove essential oil has demonstrated its effectiveness as a major packaging material for postponing the ripening process while maintaining nutritional characteristics (69).

### Processed foods

Due to alterations in lifestyle and the growth of refrigerated delivery networks, the business for pre-cooked foods (ready-to-cook processed foods) has grown recently. The quality of processed food products is deteriorated, endangering the health of customers as a result of unpleasant aromas, discoloration, stickiness, sedimentation, fumes, and altered pH. Since it has been demonstrated that adding 5% (w/w) CEO to processed food products harms their organoleptic qualities, its application has centered on using it as flavoring element with antibacterial and antioxidant characteristics (70). Food product preservation has always been a challenging undertaking. Different types of food products are preserved using different packaging materials. Recently, there has been a strong interest in using natural essential oils that are GRAS as preservatives in food items. Potential antibacterial properties exist in clove essential oil. Similar to eugenol, its active ingredient possesses antioxidant effects (71).

### Vegetables

Vegetable post-harvest degradation during transit and storage causes substantial financial losses across the supply chain (72). CEO's antibacterial qualities make it a suitable replacement for artificial fungicides by preventing fungal rotting of vegetables and harmful health impacts. Combining it with UV-C light therapy or altered packaging can increase antibacterial activity. These procedures enable efficient post-harvest destruction control and maintain the physicochemical standards of vegetables, extending their shelf life without compromising their organoleptic qualities (73). In place of sodium bicarbonate, acetic acid, and chlorine-based disinfectants, CEO is used for washing of freshly cut vegetables to lower microbial risks and increase shelf life. Moreover, CEO wash does not affect the sensory, compositional, bioactive, or color properties. To increase the marketing of vegetables with improved and long-lasting post-harvest quality followed byimproved consumer acceptance, commercial applications of CEO in conjunction with the cold storage are effective ecological option (73).

### Meat, poultry, and seafood products

When CEO is used on animal food products, it lessens the unfavorable reactions that result in the sensory qualities' loss of flavor, aroma, color, and texture (56, 74). Due to its antioxidant qualities, its antimicrobial action results in a reduction in the number of bacteria, a reduction in the ability of non-protein nitrogenous substances to degrade, and a reduction in the creation of hydroperoxide. CEO's antioxidant activities are mediated by transition metal binding, chain reaction inhibition, hydroperoxide dissolution, and free radical interaction. White shrimp, fish filets, salmon burgers, chicken patties, ground beef, and chicken breast meat have all been given the CEO label for storage in the fridge or freezer (75, 76). CEO-fortified films can prevent weight loss, lipid oxidation, water activity, change in color, and microbial growth in foods of animal origin for up to 45 days if heated and 12 days if stored at refrigeration temperature (77, 78). The meat industry has employed a number of antimicrobial treatments to decontaminate/inhibit disease-causing bacteria and increase shelf life. Antibiotics or synthetic compounds are used as these therapies. The hunt for natural sources of antimicrobials as substitute preservatives in meat products has resurfaced in response to increased knowledge of antibiotic resistance and the negative consequences of synthetic preservatives. According to studies, CEO is effective against both Gram-positive and Gram-negative germs that have been inoculated on read meat. Essential oils are a new option for natural food protection in today's market, which is centered on the usage of natural products (79).

## Potential bioactive properties of clove essential oil

**Figure 11:**
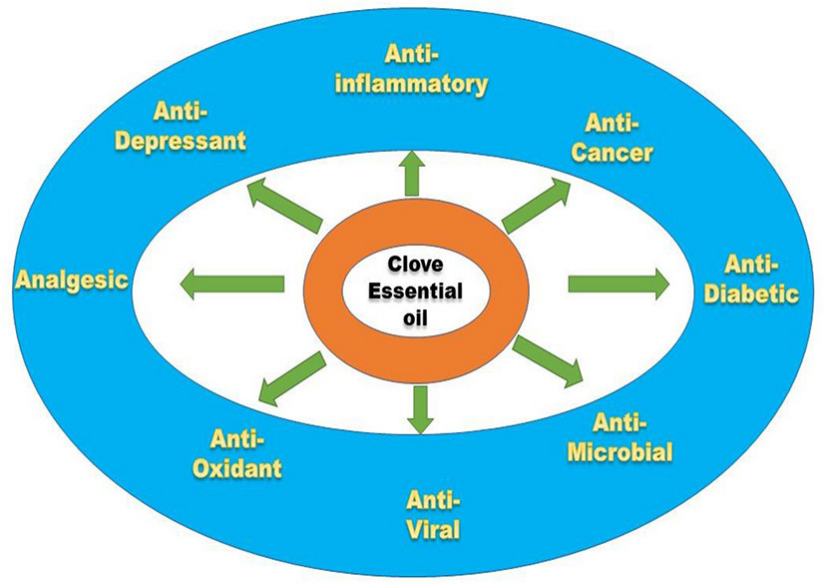
Bioactive potentials of clove essential oil (CEO).

Clove essential oil carries numerous properties of health care that includes food sector as well. Schematic representation of bioactive properties is given in Figure 11 and mechanism of actions is represented in Table 3.

**Table 3:** Bioactive properties of CEO with mechanism of action.

**S.N**.	**Bioactive Property**	**Mechanism of action**	**Reference**
	Antimicrobial activity	The clove essential oil's inhibitory zones for *S. aureus, E. coli, L. monocytogenes*, and *S. Typhimurium* were 2.83, 2.81, 2.47, and 2.22 cm, respectively. The presence and dimensions of the inhibitory zone reveal the bacteria's sensitivity to the essential oil. A sample is deemed inactive toward bacteria when the inhibition zones are smaller than 0.7 cm. Inhibitory action is satisfactory when the inhibition zone diameter is bigger than 1.2 cm. This study found that the clove essential oil had a sufficient inhibitory impact on all microorganisms tested. The literature does note a wide range of clove essential oil's inhibitory effects, though. Eugenol is thought to be responsible for the activity of clove essential oil. This substance encourages the bacterial cytoplasmic membrane to rupture, enhancing its permeability and enabling ion extravasation and the loss of intracellular proteins, both of which result in apoptosis Up to a frequency of 0.304 mg mL-1, *S. aureus, E. coli, L. monocytogenes*, and *S. Typhimurium* bacteria were inhibited by the clove essential oil. Therefore, this impact was unaffected by the properties of the membranes found in microorganisms.	(80)
	Antioxidant activity	At a level of 484.7 g mL-1, the clove essential oil's ability to scavenge DPPH was 94.86%. For the essential oil quality of 12.25 g mL^−1^, lower restriction of 28.83 and 22.13% for the hydroxyl and nitric oxide radicals, respectively, were seen. Clove essential oil's significant DPPH scavenging activity can be attributed to the synergistic effects amongst phenolic components, even at low doses. The lower inhibition values for hydroxyl and nitric oxide radicals, however, may be the result of the phenolic compounds' limited interaction with these extremists.	(80)
	Treating Dental Erosion and pain	Due to the potent chemical eugenol, it is still a well-liked remedy today. Eugenol is an organic sedative. Although clove essential oil is excellent in treating pain, there isn't enough proof to say that it can also efficiently eradicate the bacteria that are the source of the issue. Some acidic foods and beverages have been shown to decalcify (degrade) tooth enamel. One study indicated that the eugenol in clove oil, when applied topically, may prevent or lessen the symptoms of dental erosion. The advantages of clove oil as a therapeutic or preventative ointment for dental enamel erosion, however, require more study.	(81)
	Antiviral activity	A strong antiviral agent is clove. At a concentration of 10 g/ml, eugenin, which was extracted from clove buds, exhibited antiviral action against with the Herpes Simplex virus.	(81, 82)
	Anti-inflammatory activity	The main chemical in clove's volatile oils, eugenol, has anti-inflammatory properties. According to animal research, adding clove extract to diets that already contain a lot of anti-inflammatory ingredients (such as cod liver oil, which has a high level of −3 fatty acids) has a synergistic impact. Kaempferol, rhamnetin, and -caryophyllene are only a few of the flavonoids found in clove that also contribute to its anti-inflammatory and antioxidant properties 26. The anti-inflammatory effects of Eugenia caryophyllata essential oil were comparable to those of etodolac at 0.025 and 0.1 ml/kg and indomethacin at 0.05 and 0.2 ml/kg doses.	(81)

### Antioxidant

*Eugenol, eugenyl acetate*, and *-humuleneand –caryophyllene*are antioxidant chemicals found in CEO that shield cells from free radical damage. The existence of ROS molecules has been linked to illnesses like Alzheimer's disease, cancer, atherosclerosis, and Parkinson's disease (83). CEO has demonstrated anti-radical action and lipid peroxidation inhibition. The antioxidant action of eugenol is due to the hydroxyl group present on the aromatic ring. The phenolic compounds impede the oxidative process by transferring electrons or hydrogen atoms to free radicals and neutralizing them (84). The most plentiful and potent component of cloves is eugenol, which is also known to have neuroprotective qualities. It is a well-known antioxidant and MAO inhibitor. Eugenol is well known for its ability to neutralize free radicals, suppress the creation of reactive oxygen species and nitrogen, boost cyto-antioxidant capacity, and safeguard the functionality of microbial DNA and proteins. Eugenol can also aid in the removal of harmed molecules, the repair of oxidative damage, and the prevention of cancer-causing mutations. It has been suggested that eugenol's structure, which enables it to repair phenoxy radicals by accepting given hydrogen atoms, is what gives it its antioxidative potential (85).

### Antiviral

CEO has demonstrated antiviral action against herpes simplex types 1 and 2, influenza A virus, and Ebola. According to recent research eugenol derivatives can reduce the West Nile Virus's activity, making them a potentially effective treatment for flaviviruses like dengue, Zika, and yellow fever (86). Due to its ability to lower virus replication, eugenol can also act as potential inhibitor of the early stages of HIV-1 infection. Eugenol can also boost lymphocyte synthesis, therefore its ability to promote lymphocyte growth may be what gives it its anti-HIV-1 effect (87). A replacement for the human norovirus, the CEO has shown antiviral effectiveness against feline calicivirus. Because of this, any viral load that may be present is eliminated when fruits and vegetables are washed using CEO. Furthermore, the inclusion of the CEO in cleaning wipes enables surface cleansing. More than moroxydine hydrochloride, CEO has been proven to boost tomato plants' resistance to the tomato yellow leaf curl virus (20).

### Antimicrobial

CEO has demonstrated broad-spectrum pathogen-inhibitory action. The -OH groups in the primary chemical composition's meta and ortho locations, respectively, have been linked to the antibacterial action. The cytoplasmic membrane of microbial cells can interact with these functional groups. Due to CEO's lipophilic characteristics, it can pass across cell membranes. When CEO interacts with fatty acids, polysaccharides, and phospholipids, the integrity of cell membrane is lost, contents of the cell leak out, and the proton pump is interfered with, which results in cell death (84). CEO can prevent the growth of yeast, Penicillium, C. albicans, Aspergillus (*A. flavus, A. ochraceus* and *A. parasiticus*), Gram-negative bacteria (*E. coli, Klebsiella pneumoniae, Salmonella*, and *L. monocytogenesand Erwinia carotovora*), Gram-positive bacteria (*Streptococcus, S. aureus*, and *L. monocytogenes*) (20). It has been suggested that the hydroxyl group on eugenol inhibits protease, histidine carboxylase, and amylase activity in Enterobacter aerogenes by binding to them. Similar to this, eugenol has been discovered to possibly impede the activity of membrane-bound ATPase in Listeria monocytogenes and Escherichia coli. Additionally, it has shown how traditional antimicrobials work together in a synergistic manner. Furthermore, it is thought that eugenol might create intracellular reactive oxygen species (ROS), which can kill cells by preventing them from growing, rupturing their membranes, and destroying their DNA (85).

### Anti-inflammatory and wound healing

In many pathophysiological situations, including hypertension, diabetes, and neurodegenerative and cardiovascular disorders, inflammation and oxidative stress are closely connected processes (88). CEO and eugenol have anti-inflammatory characteristics similar to diclofenac gel, decreasing inflammation from 60 to 20% after 3 h. Similarly, CEO-treated rats with generated wounds experienced a dramatic shrinkage of more than 95% within the first 15 days. These findings show that CEO-treated mice saw wound healing comparable to that of neomycin-treated animals, which is currently utilized to reduce inflammation and speed up wound healing. As a result, it is possible to prevent both the acute and chronic negative impact of synthetic antibiotics, particularly if they are used regularly (23).

### Anticancer

Cancer prevention and co-treatment have both benefited from the usage of CEO's eugenol, humulene, and caryophyllene components, havingantitumor and cytotoxic effects. According to certain findings, EOs may lessen the chemotherapeutic side effects of vomiting, nausea, appetite loss, and weight loss. Since the formation of ROS particularly stimulates signaling pathways and results in the growth of cancers by controlling cell proliferation, angiogenesis, and metastasis, the anticancer action is mostly related to the anti-inflammatory and antioxidant activity (83). CEO has been evaluated against several cancer types, including colon, lung, breast, pancreas, leukemia, cervical, and prostate cancers (89). In both *in vivo* and *in vitro* experiments on triple-negative breast cancers, eugenol was also discovered to increase the cytotoxic and pro-apoptotic action of cisplatin, a cytostatic medication. Eugenol is thought to increase cisplatin's ability to suppress breast cancer stem cells by decreasing the activity of aldehyde dehydrogenases (ALDH), ALDH-positive tumor starting cells, and the NF-B signaling pathway. These findings imply that eugenol and cisplatin combination therapy may be a successful treatment for triple-negative breast cancers (85).

### Anesthetic

CEO is acknowledged as a safe anesthetic in vertebrates and invertebrates at low concentrations. Without changing the responsiveness to external stimuli, it causes anesthesia more quickly, has a quick reflex recovery, and has a low mortality rate. According to recent research, CEO and eugenol diminish corneal sensitivity in rats in a manner similar to lidocaine. The maximal amount and length of anesthesia rely on the exposure period and concentration, which vary based on the chemical. In Nile tilapia, cardinal tetra, ringed cichlid, and angelfish, CEO effectively produces anesthesia, altering swimming ability and balance as well as reducing the reaction to environmental stimuli until full immobility. The amount of time needed to reach full anesthesia decreases with dose concentration. Additionally, when recovering from anesthesia, there are no negative effects of CEO based on the dose and exposure period (20).

### Pharmaceutical mechanism of action

The dried flower buds of the clove plant are used to extract clove oil. It has historically been used as a scent or as a spice to flavor meals. Additionally, topical analgesics contain it. It has been demonstrated that clove oil has some antibacterial effects. Clove has greater antioxidant and antibacterial activity than many other fruits, vegetables, and spices. Although it originated in Indonesia, clove is now grown all throughout the world, especially in the Brazilian state of Bahia. This plant has a lot of promise for use in medicinal, cosmetic, food, and agricultural applications because it is one of the richest sources of phenolic chemicals like eugenol, eugenol acetate, and gallic acid. Interestingly, clove oil has shown promise in studies looking at its potential for treating vaginal candidiasis and neuropathic pain. Clove oil is classified by the FDA as generally regarded as safe (GRAS) for use in food additives or dental cement.The phenol “eugenol,” which is contained in clove oil in concentrations up to 85%, is the main component. Escherichia coli, Staphylococcus aureus, and Pseudomonas aeruginosa are all germinated by clove oil. The synthesis of prostaglandins is hypothesized to be inhibited by clove oil, which lessens painful symptoms. The primary component of clove oil, eugenol, is said to have anticancer properties. In one study, DNA fragmentation and the production of DNA ladders in agarose gel electrophoresis were signs of death in HL-60 cells treated with eugenol. It was shown that eugenol generated reactive oxygen species (ROS), which then caused mitochondrial permeability transition (MPT), decreased levels of the anti-apoptotic protein bcl-2, caused cytochrome c to be released into the cytosol, and ultimately caused apoptotic cell death (10).

## Clove essential oils in edible coatings

Edible coatings have established themselves as a successful primary packaging material for postponing the ripening process while preserving nutritional qualities. Additionally, it was found that adding active ingredients like essential oils to edible coatings considerably increased their effectiveness. The utilization of CEO in nanoemulsion for the active delivery of their antibacterial and antioxidant properties. By safeguarding and sustaining the more shelf stability, this union seeks to maintain the freshness of fruits (69). Active packaging is divided into bioactive and chemo activecategories depending on the additives included in the film. Chemically reactive packaging affects the gaseous environment and food products' chemical make-up inside a pack. It consists of moisture control systems, modified environment packing, and ethylene scavengers. The growth of many microbes may be prevented by bioactive packaging, which has antioxidant and antimicrobial chemicals that interact with biological molecules. Eucalyptus globulus essential oil in chitosan was employed in a study by Azadbakht et al. (90) to investigate the antibacterial activity of the packaging of sliced sausages. The outcomes resulted that elevating CEO concentration might enhance the log reduction value. The use of essential oils in active packaging's, likeclove oil, cinnamon oil, and thyme oil, is possible in the form of films and coatings. Films are often thin sheets that are prepared in advance for use as covers, wraps, layer separators, or food packaging. Contrarily, coatings are characterized as films which can be applied to an edible product's surface (91).

**Figure 12:**
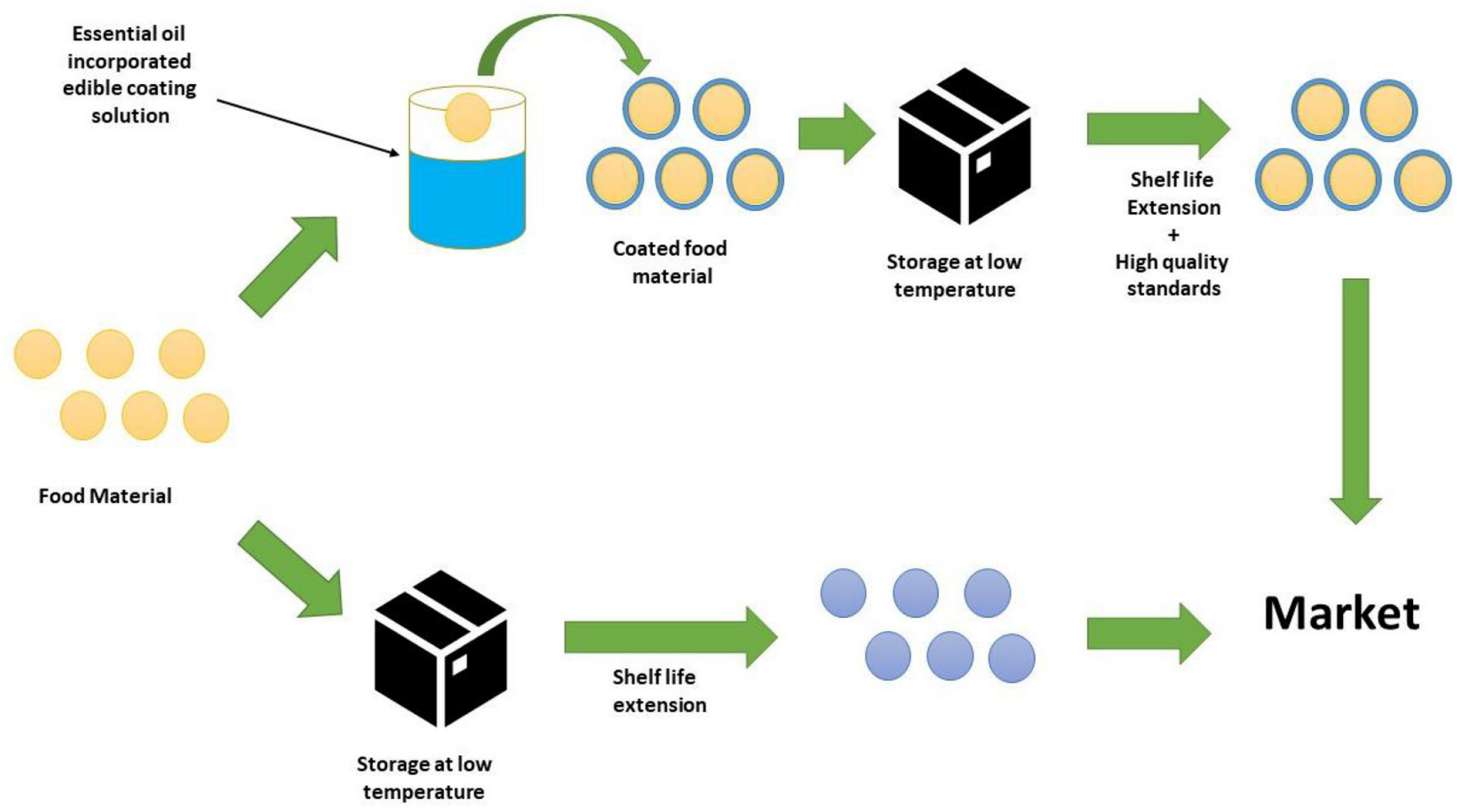
Coating with essential oil incorporated edible coating material.

The physicochemical characteristics of potato starch films that had been cast with various amounts (0.5, 1.0, 1.5, 2, and 2.5%) of clove essential oil (CEO) were examined. The addition of CEOs to the films increased the thickness of film, which had an impact on the films' opacity and raised the turbidity and yellowness of the films. The moisture content of the films decreased as a result of the essential oil's incorporation becoming more concentrated. These enhanced the starch films' water resistance and made them desirable for use in food packaging. As compared to the control sample, the use of CEO significantly decreased the rupture force and rupture distance of the films. According to the thermal analysis, adding CEO up to 1% stabilized the films. The generated multicomponent films could be a suitable material for creating practical food packaging because they have better physical qualities (92). Diagrammatic representation of edible coating mechanism incorporated with CEO is given in Figure 12.

Active films have been created with various instances of essential oils and their chemical compounds. For the packaging of sliced sausages, chitosan films with Eucalyptus globulus essential oil were created. These films have a strong potential to inhibit antimicrobial effect and restrict the spread of food-borne contamination in food systems (90). Another investigation by Perdones et al. (93) showed that the slower respiration rate of strawberries treated with chitosan-based coatings containing lemon essential oil significantly delayed the ripening process. It was also shown that the flavor of lemon essential oil did not affect the organoleptic qualities of strawberries after 7 days of storage. The chemical makeup of utilized essential oil was cited as the cause of these newly developed packaging systems' antibacterial action (93).

Apple pieces coated with CMC and ascorbic acid showed a noticeably reduced tendency to brown (94). The apple slices treated with CMC in conjunction with CaCl_2_ and ascorbic acid effectively preserved their acidity, pH, firmness, and ascorbic acid content; however, POX and PPO activity significantly decreased and SOD activity significantly increased. To improve the quality of papaya held at 12°C for 28 days, a mixture of 2% ginger oil and 10% gum Arabic was effective. Anthracnose was significantly inhibited by the therapy. The fruit's sensory qualities were not altered, but its firmness, acidity, and weight were preserved. *Mentha piperita L*. or *Mentha x villosa* was added to chitosan. *Aspergillus niger, P. expansum,B. cinerea*, and *Rhizopus stolonifer* were all successfully combatted by Hud's essential oils, which also protected the texture, color, titratable acidity and total soluble solids, of cherry tomatoes without changing their sensory qualities (95). It has been shown successfully to add essential oils to edible films to act as antibacterial and antifungal agents. Adding rosemary oil to HPMC did not appreciably change the film's mechanical characteristics. HPMC, however, demonstrated decreased percent elongation at break at high oil concentrations at a concentration of 6%. At 1% oil content in 6% HPMC, the films' water vapor permeability also increased.

Oregano essential oil in alginate at a concentration of 1%had an impact on the films' thickness and tensile characteristics. The films' water vapor permeabilityandtensile strength, however, both decreased. Additionally, *S. aureus, L. monocytogenes, S. enteritidis*, and *E. coli*, could all be shown to have an antibacterial effect. The impact of oregano essential oil added to chitosan-based and fish gelatine films was also studied by Hosseini et al. (96). However, *E. coli* was discovered to be the most resistant. The authors discovered that films having oregano essential oil prevented the growth of *S. aureus*. Additionally, films that contained oregano essential oil at the concentration of 0.4% (w/v) exhibited greater plastic deformation. However, the elasticity of bio composite films increased when more oil was added to them (e.g., 0.8 and 1.2% w/v). It was discovered that adding oregano essential oil drastically reduced the tensile strength and elastic modulus. When oregano essential oil was incorporatedinto the films, water solubility was dramatically enhanced. The films' transparency has also improved (96).

## Classification of edible coatings

As CEO cannot be used directly to give a protective covering to the food material, scientists have used edible coatings as a carrier to deliver the bioactive properties of CEO or any other essential oil. The development of edible coatings and films from different sources has been the subject of extensive research throughout the years. Interestingly, applying various films or coatings has produced a range of outcomes because of the differences in their mechanical and structural qualities. Only a small number of recent advancements in this area have been critically examined here. Classification of coating materials with their mechanism of action is represented in Table 4.

**Table 4:** Types of coating materials with mechanism and effects.

**Coating material**	**Types**	**Effects/Mechanism**	**References**
Hydrocolloids	Polysaccharide-based edible coating	These coatings are applied on minimally processed or fresh fruits and vegetables, by creating modified atmospheric conditions to decrease their respiration rate. These coatings have enhanced mechanical handling property and additives carrying capacity. These coatings have better gas barrier properties, leading to the desirable modified atmosphere that extends the shelf life of fruits and vegetables without formation of anaerobic conditions.	(97)
	Protein-based edible coating	These coatings consist of better barrier properties for oil, aroma, and oxygen and it increases strength, due to their tightly packed hydrogen-bonded structure. These coatings are not good barriersto water vapor due to their hydrophilic nature but they consist of better organoleptic and mechanical properties	(98)
	Casein, whey and zein protein	Casein is mostly used to prepare emulsion as it is amphipathic and contains hydrophilic and hydrophobic ends. Casein is also used for edible coating as casein edible coatings can be formed easily. Zein films and coatingshavebetter barrier properties to water vapor, about 800 times higher than other wrapping films and edible coatings. All properties of zein coatings depend upon thickness of coating.	(99)
Lipid-based edible coating	Waxes	Lipids possess good water barrier capacity. Wax coatings contain better moisture barrier properties as compared to other lipid-based coating and non-lipid coating. Combination of lipid and polysaccharides, proteins are used in coating material to enhance their barrier properties.	(100)
	Lacs		
	Acetylated glycerides		
	Fatty acids and alcohols		
	Cocoa-based material		
Composites-Based Edible Coating	Bilayer composites	Composite coatings contain a combination of polysaccharides, protein, and lipid-based material. It is used to increase mechanical strength, and gas and moisture barrier properties of edible films and coatings.	(101)
	Conglomerates		

### Polysaccharide based coatings

The majority of seasonal fruits and vegetables are collected in large quantities and kept in the right conditions. To market them at their highest quality and minimize post-harvest losses, this is done (102). The main issue with fruit transportation or trade is its short shelf life. If appropriate preservation measures are not used, the respiration process continues even after harvest, resulting in lower shelf life. Because of their convenient availability, low cost, better barrier characteristics against respiratory gases, etc., polymeric films (plastics) are frequently used in food industry to pack fresh fruits and vegetables. However, because these packaging materials are not biodegradable, they harm the environment. As a result, the food industry is now focused on environmentally friendly biodegradable packaging including edible coatings and films (103).

**Figure 13:**
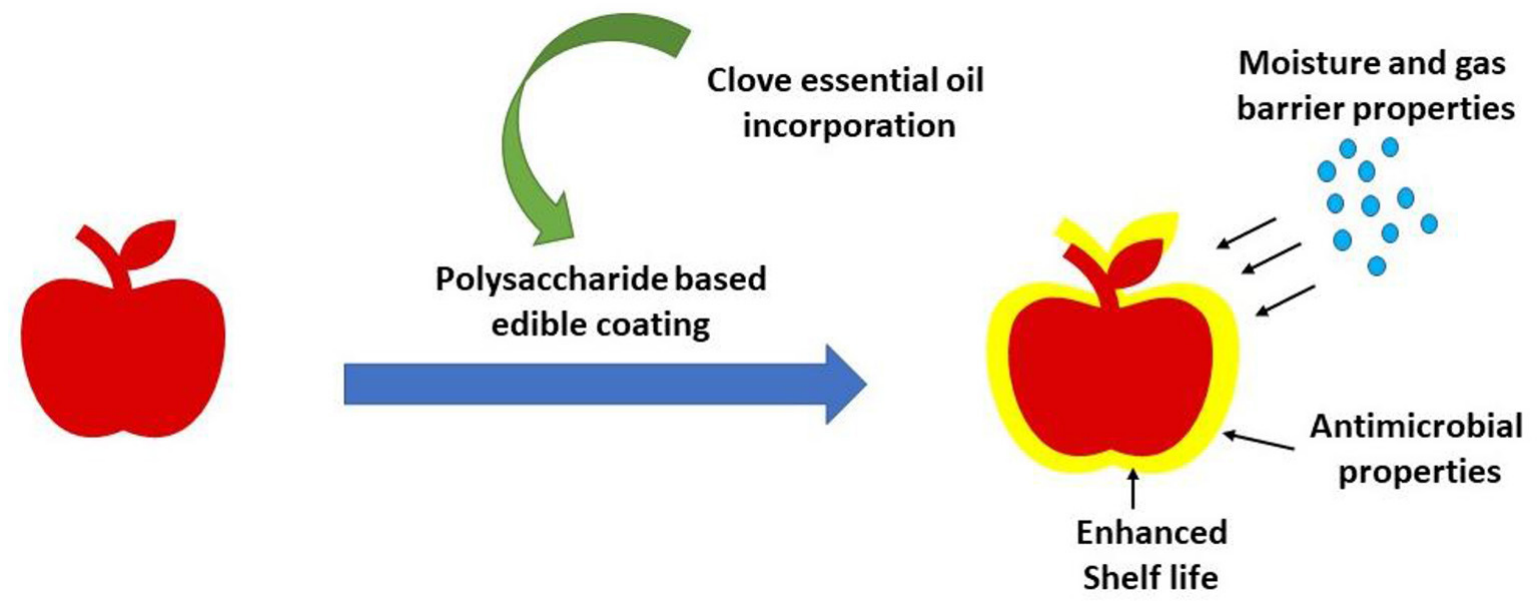
Application of edible coating on fruit.

The polysaccharide component known as alginate is often generated from brown algae and soil bacteria and used in food industries as gelling agent. Alginate is appropriate for use in the creation of edible coatings and films because it creates a gel-like structure when divalent cations, typically calcium, are present (104). The 1, 4 linkages of D-mannuronic acid and L-guluronic acid residues make up the commercially available alginates. Freshly cut pineapples have been examined with an edible covering made of alginate (105). When compared to control fruits kept at a chilled temperature, the shelf life of the freshly cut Fuji apples covered with alginate coating increased by almost three times. Due to its stronger tensile strengthanddecreased water vapor permeability, the alginate coating on the pears' fruits proved successful in minimizing changes in firmness, weight loss, total soluble solids, pH, and color. At a temperature of 25°C, this alginate coating (2%) preserved the physical and chemical characteristics of the pear for up to 15 days (106). Visibility of edible coating functioning on fruit is shown in Figure 13.

#### Carbohydrate-based coatings

**Figure 14:**
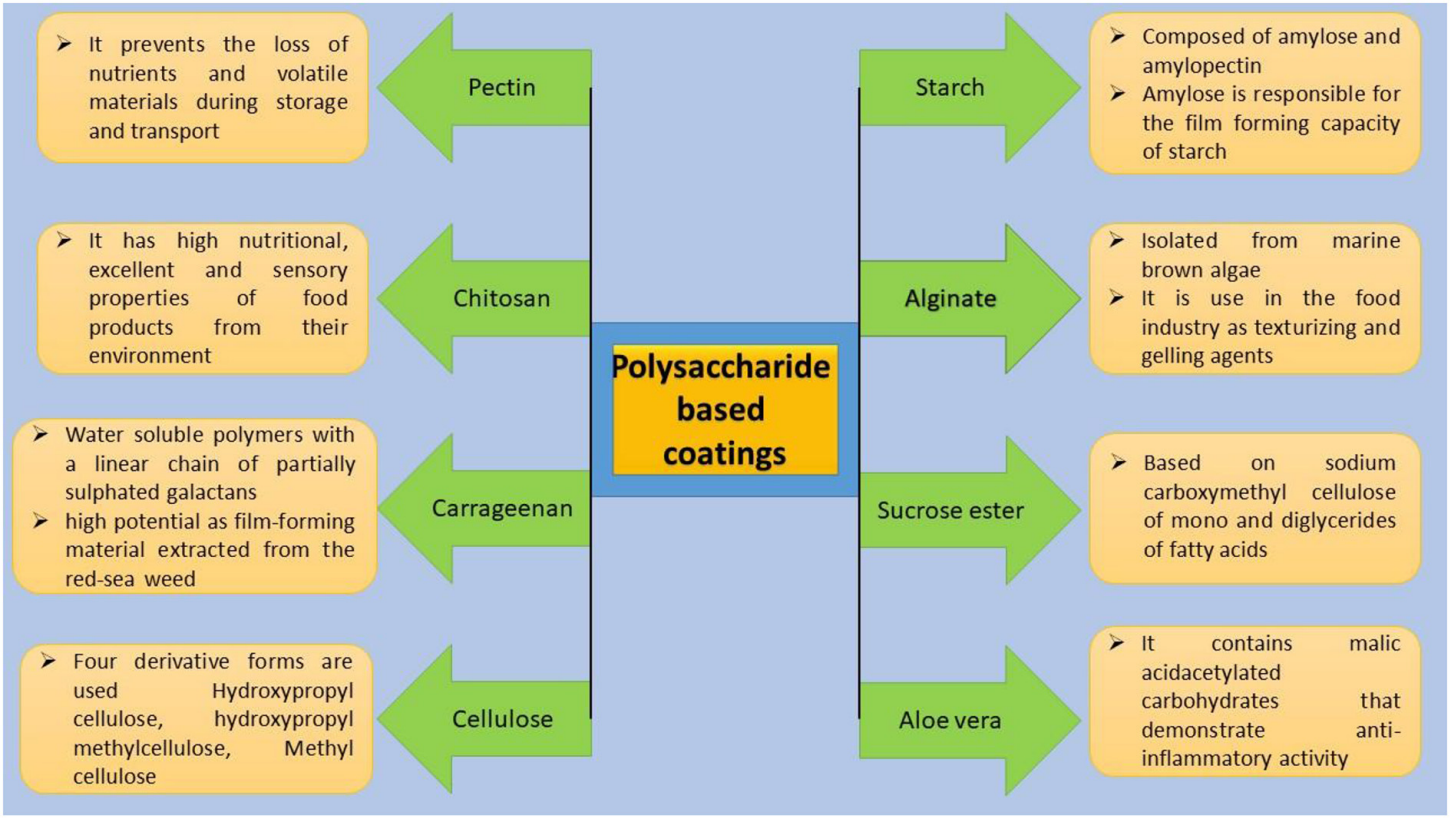
Classification of polysaccharide based edible coatings.

As thickeners, stabilizers, gelling agents, and emulsifiers, carbohydrates are employed in food systems. polymeric films possess minimal or no permeability to gases, but resistance to the transfer of water vapor. These coatings are used to prevent some plants from losing moisture foodstuffs during temporary storage. Carbohydrate Films can be used for surface polishing and product separation within the packaging. Further classification of polysaccharide based edible coating is depicted in Figure 14.

### Protein-based coatings

For use on freshly cut mango fruit, Sharma et al. (107) created and investigated the impact of bio composite edible coatings made of crosslinked-sesame protein and mango puree. During the 15-day storage period, a significant impact of the bi-layer coatings enhanced with calcium chloride and guar gum was seen. Fresh-cut mango fruit's shelf life was successfully extended by all coverings. Sesame protein films have superior barrier qualities compared to those made with peanut, soy, mung bean and whey proteins. To extend the shelf life of freshly cut pineapple kept in polystyrene trays for 15 days at 5°C, Sharma et al. (108) developed bilayer coverings from native sesame protein and organic acid crosslinked sesame protein. In comparison to uncoated samples, the application of bilayer coatings based on native/cross-linked sesame protein and calcium chloride-pineapple juice improved firmness, titrable acidity, driploss, and color. Over 15 days of storage, coatings had a minimal impact on total and reducing sugars. Also indicating controlled microbial growth during storage is the limited total plate count for all coated samples compared to untreated samples (108).

### Lipid-based coatings

Lipids are effective moisture-retention barriers because of their superior hydrophobic qualities. By producing a high sheen and reducing respiration, lipid-based coatings have demonstrated their effectiveness in increasing shelf life and enhancing the appearance of fruits and vegetables. A variety of lipid materials, including natural waxes, acetylated monoglycerides, and surfactants, can be used to create lipid-based coatings. To increase the shelf life of guava fruit, Olieveira et al. (86) created biopolymeric films using cassava starch, maize starch, beeswax, and gelatin. The highest results in terms of water vapor transmission rate were shown in biofilms containing 10% beeswax (WVTR). Beeswax was added, and the elasticity increased by 80% but the solubility dropped by 15%. A physicochemical investigation revealed that the use of coatings prevented weight loss and enabled the fruit to ripen enough over 15 days of storage. Fruits with coatings are more palatable than uncoated fruits, according to sensory evaluation (86). Numerous research works (109–114) proved that edible coating is the effective technology for preservation of fruits and vegetables.

## Conclusion

CEO is a food ingredient that the FDA has generally deemed to be safe. The phenological stage, agroecological circumstances, processing conditions, pre-treatment, and extraction techniques all have a direct influence on the chemical composition of the CEO. The bioactive substances responsible for their beneficial effects on health can be extracted selectively thanks to new techniques. The primary volatile chemicals having antioxidant, antibacterial, anti-inflammatory, analgesic, antiviral, and anticancer effects are eugenol, -caryophyllene, -humulene, and eugenol acetate. The CEO's antibacterial and antioxidant properties have promoted their use in the food industry's poultry, meat, vegetables, seafood, dairy goods, and edible coating films. For possible use in the therapy of various disorders, more research is required to determine the functions of the primary constituents in the diverse biological processes. Furthermore, it's important to figure out whether these elements work in harmony or conflict with one another. The use of CEO in the food sector also has to be studied, particularly how it can act as an antioxidant or antibacterial agent without impairing food's flavor, color, or texture. There aren't many reports on CEO encapsulation's impact on the key physicochemical and biological characteristics. The impact of encapsulation methods on absorption, solubility, bioavailability, and shelf life by preventing degradation (photo, oxidative, or thermal), as well as its impact on organoleptic qualities, still needs further investigation. An increasingly fascinating area of research is the creation of edible coatings or films using nanotechnology, which could lead to the creation of nano capsules by adding active substances. The edible coatings or films would have improved stability and a better ability to release the active ingredients thanks to the nano components. To comprehend the mechanisms of action of various active components with various edible films and coatings and their impact on the mechanical and sensory qualities, more research is required.

## Future perspective

Even if CEO is commonly used and consumed, there may still be some uncharted territory. For possible use in the therapy of various disorders, more research is required to determine the functions of the primary constituents in the diverse biological processes. Furthermore, it's important to figure out whether these elements work in harmony or conflict with one another. The use of CEO in the food sector also has to be studied, particularly how it can act as an antioxidant or antibacterial agent without impairing food's flavor, color, or texture. There aren't many reports on CEO encapsulation's impact on the key physicochemical and biological characteristics. The impact of encapsulation methods on solubility, absorption, bioavailability, shelf life by preventing degradation (photo, oxidative, or thermal), as well as its impact on organoleptic qualities, still needs further investigation.

## Author contributions

VK, RSh, RSi, and MT: original manuscript writing. AD, RP, and AR: original manuscript writing and reviewing. All authors contributed to the article and approved the submitted version.

## Funding

This study was supported by a grant from the Romanian National Authority for Scientific Research and Innovation, CNCS—UEFISCDI, project number PN-III-P2-2.1-PED-2019-1723 and PFE 14, within PNCDI III.

## Conflict of interest

The authors declare that the research was conducted in the absence of any commercial or financial relationships that could be construed as a potential conflict of interest.

## Publisher's note

All claims expressed in this article are solely those of the authors and do not necessarily represent those of their affiliated organizations, or those of the publisher, the editors and the reviewers. Any product that may be evaluated in this article, or claim that may be made by its manufacturer, is not guaranteed or endorsed by the publisher.
